# Tied Migrant Labor Market Integration: Deconstructing Labor Market Subjectivities in South Africa

**DOI:** 10.3389/fpsyg.2022.806436

**Published:** 2022-04-12

**Authors:** Farirai Zinatsa, Musawenkosi D. Saurombe

**Affiliations:** ^1^Department of Development Studies, University of the Free State, Bloemfontein, South Africa; ^2^Department of Industrial Psychology and People Management, University of Johannesburg, Johannesburg, South Africa

**Keywords:** tied migrant, female, labor market integration, intersectionality, South Africa

## Abstract

The South African labor market is characterized by a high degree of inflexibility and complexity which poses significant challenges for both indigenes and migrants looking to be integrated into the labor market. These challenges are likely to be more poignant for international migrants as they face additional barriers owing to a chronically high employment rate, xenophobic sentiments, and racial exclusion. For female tied migrants, gender bias, expressed through migration policies and legislation, adds yet another layer of complexity to long-term aspirations of settling in South Africa. How well tied migrants fare in the South African labor market is an important matter for consideration. Using an intersectional approach and the theory of governmentality, this study sought to deconstruct the labor market subjectivities of tied migrants in South Africa. This paper used a qualitative approach, with a narrative and interpretivist research paradigm, on female tied migrants from Sub-Saharan Africa who either accompanied their husbands or followed them to South Africa in a process of family reunification. Although 13 interviews were carried out in total, as part of a broader study, the narratives of six participants were included in this study, to zone in on labor market experiences. The study found that despite their high human capital, tied migrants are not likely to be well integrated into the South African labor market. Their inequality in the South African labor market was attributed to their gender, ethnicity, race, migrant status and locality and various intersections thereof through which they are subjected to informality, immobility and precarity.

## Introduction

Labor market participation is one of the strong predictors of long-term integration into society and a key instrument through which migrants can make monetary contributions to the host society (Konle-Seidl and Bolits, 2016). For migrants arriving in South Africa in search of better opportunities for socioeconomic wellbeing however, the South African labor market poses significant challenges for integration.

Like the status quo in other parts of the world (Ballarino and Panichella, 2017; Ressia et al., 2017), research in South Africa suggests smoother transitions into the labor market for male migrants in comparison to their female counterparts who are faced with formidable barriers to overcome (Mbiyozo, 2018; Souza and Flippen, 2020). While a substantial amount of research has been conducted on the labor market, skilled female migrants, particularly those emigrating in the context of family, remain largely obscured in extant literature on labor migration. This could be attributed to the fact that the dominant image of the skilled migrant is male, and the fact that family migration is generally regarded as a social process (Bailey and Mulder, 2017). For instance, accompanying spouses arriving in the context of family in South Africa are legally designated the status of the dependent, which makes it illegal for them to work, study or conduct business (Department of Home Affairs, 2017).

The literature shows that skilled and tied migrants, however, remain agentive (Riaño, 2012; Bailey and Mulder, 2017; Grigoleit-Richter, 2017; Kõu and Bailey, 2017; Föbker, 2019; Di Martino et al., 2020) and seek ways to subvert governing technologies that keep them excluded from the labor market. Owing to the complexity of the South African labor market, some of the previously mentioned authors argue that it is inadequate to focus on the binary of employed or unemployed. This suggests the need for labor market integration further.

Little is known about how skilled female migrants who emigrate in the context of family migration, fare in the labor market in South Africa. Using an intersectional lens and the theory of governmentality this article explores the labor market experiences of skilled female migrants who emigrate to South Africa in the context of family migration. In doing so, it seeks to contribute to the literature on the transitions of skilled migrants who remain largely understudied in the global South context.

## A Brief Literature Review

### Intersectionality

Intersectionality has traditionally been utilised as a heuristic device to unlock multiple layers of social stratification (Ressia et al., 2017; Kaushik and Walsh, 2018; Atewolougun, 2019) that female migrants face in countries of destination. First coined by Kimberle Crenshaw, the term intersectionality was used to explicate how access to the labor market by black American women was influenced by intersections of gender, race, and ethnicity (Crenshaw, 1989, 1991). Crenshaw (1989) emphasized that the marginalization these minority women faced was mutually constituted. An intersectional lens, therefore, provides for simultaneous attention to different social structures as they give rise to different social positions and subsequently lived experiences (Anthias, 2012; Ressia et al., 2017). While particular attention has been paid to this trinity of disadvantage, over the years, new categories of intersection such as sexuality, and disability/health have been proposed (Amelina and Lutz, 2021).

Critically, the use of an intersectional approach can help understand the “interconnectedness, interdependencies and mutual co-construction of key categories of social marking and positioning” (Amelina and Lutz, 2021, p. 57). Applying an intersectional lens to studies on skilled migration has several advantages. According to Morokvasic (2014, p. 357), an intersectional lens helps to illuminate “who works and where, whose work is acknowledged as work, whose is invisible and unrecognized,” and how life is destabilized and negotiated in the migration process. In this way, it can help to comprehensively determine the issues that impact on settlement and integration including issues of identity, inequality/privilege and to better target those in need of integration services (Kaushik and Walsh, 2018). One of the major criticisms of intersectionality, however, is that it has not been well operationalized (Ressia et al., 2017; Atewolougun, 2019).

### Intersectionality and Labor Market Integration

Research on intersectionality and the labor market reveals that the labor market experiences of tied migrants are shaped by various identities (Bailey and Mulder, 2017; Fobker and Imani, 2017; Ressia et al., 2017). Because of these various identities including, race, ethnicity, and migrant status (Kaushik and Walsh, 2018; Mbiyozo, 2018), tied migrants are subject to differential treatment in host countries.

For accompanying women arriving in the context of family migration several factors constitute what may be regarded as primary forms of discrimination. Compared to other migrant women who experience a “double earnings penalty” because of their categorization as immigrants and as women (Ballarino and Panichella, 2017), women arriving as family members are likely to suffer triple penalties based on their gender, immigration status and migration background (Mbiyozo, 2018). Their condition, therefore, would not be much different from refugees and asylum seekers who are among the most marginalized migrants and are subjected to the highest levels of discrimination.

Evidence shows how on account of gender norms, migrant status, and ethnicity, tied migrants commonly face discriminatory norms in the countries in which they choose to settle (Bailey and Mulder, 2017; Grigoleit-Richter, 2017; Ressia et al., 2017). Discrimination directly impacts the labor market integration outcomes of immigrant women. This discrimination can take on various forms such as ethnicity, race, class, religion, and gender intersections to create barriers to LMI for immigrant women (Wojczewski et al., 2015; Korteweg, 2017; Kesler and Safiz 2018; Chinyakata et al., 2019). Wojczewski et al. (2015), for example, highlight how the discrimination of Black African migrant care workers based on gender and race is more poignant than for other migrant races. Racialized minorities typically face the greatest discriminatory barriers. For instance, Kesler and Safiz (2018) found that Pakistani and Bangladeshi women realize much higher penalties in labor market integration than other immigrant women groups in France. The discrimination that skilled women immigrants face can be imperceptible, but it often results in barriers to advancement in the workplace (Grigoleit-Richter, 2017). However, discrimination in the labor market may manifest at any stage in the labor processes, including in hiring, promotion, retrenchment, work assignment, promotion, or retirement (Cooke, 2007). Wage differentials are also common in virtually all western European countries, where it is common for immigrants from non-Overseas Economic Cooperation and Development (OECD) countries to significantly lag with respect to wages.

Because of their gender, tied migrants are likely to be accompanying partners (Cooke, 2007; Fobker and Imani, 2017). They are also likely to face deskilling and downward occupational mobility, domestication, and their subsequent care responsibilities are likely to make it very difficult for them to be integrated into the workplace (Fobker and Imani, 2017; Ressia et al., 2017).

Othering practices based on ethnicity also extend to the workplace (Yeoh and Lam, 2012; Grigoleit-Richter, 2017) where they can produce direct impacts for the career trajectories of skilled migrants. A study by Grigoleit-Richter (2017) for example, showed how ethnic based discrimination resulted in a significant impact on the professional identity of skilled migrants in Germany, and the slowing down of the transfer of their cultural capital.

Integration into the labor market is not without its challenges and tied migrants are likely to face further disadvantage based on their gender, migrant status, ethnicity etc. (Bailey and Mulder, 2017). Tied migrants also typically experience exclusion from the labor market or being employed below their skill level. Skills that are not utilised fully, represent brain waste. In this regard, Beukes et al. (2017) warn that the possible underutilization of skills and qualifications in the South Africa labor market could exacerbate structural problems within the South African economy.

According to Atewolougun (2019, p. 4) “embedded within each socially constructed category, is a dynamic related to power and power interrelations” and this makes it imperative for intersectional analysis to pay attention to power. As a result of power relations, people may experience unequal treatment in the determination, application, and implementation of rules (Collins and Bilge, 2020). In this regard both intersectionality and governmentality are key considerations as it relates to labor market integration of tied migrants in the South African labor market.

The migration legislation in South Africa sets up a different application of treatment for tied migrant women arriving in the context of family. Their identity as dependents is assigned through legality and as a result this makes it significantly difficult for them to access the labor market even when they are in possession of appropriate qualifications and work experience. This can be characterized as an explicit gender bias which plays out in legislation and migration policies to the detriment of female international migrants (Dinbabo and Nyasulu, 2015). The determination of tied migrants’ transition into the host country as a social process, belies their aspirations to re-establish their careers. The application of power therefore relates to their identity as married women, migrants, and relates to the acknowledgement of their skills. In this regard, power relations are an important consideration not only for access and integration but also long-term career outcomes.

### Power and Subjectivities

As a critical theory, intersectionality, like governmentality, recognizes that power works by making various knowledges available and these ultimately shape identity, person, and self (Rose et al. 2006; Liversage, 2009; Dean, 2010; Ho, 2017). Critically, the shaping of subjectivities subsequently shapes lived experiences. Subjectivities, however, are not totalizing and can be challenged and resisted at a micro level (Hudson et al., 2019). This notion of resistance or counter-conducts encourages the interrogation of the “flows of power in the opposite direction,” i.e., bottom-up (Ball and Olmedo, 2013, p. 86). This is at the center of the notion of freedom or agency, and the bi-directional nature of power (Death, 2016).

“The will not to be governed thusly, like that, by these people, at this price” (Foucault, 2007, p. 75) however, is not foregone conclusion. This is reflected in that while tied migrants may want to be integrated into the labor market (alternate subjectivity) they may be prevented from doing so due to deeply entrenched practices of ethicized discrimination. This inability to subvert all governing technologies in the host society is reflected in the fact that migrants may need to be assisted to integrate into society through specific integration programs. This does not suggest the absence of resistance but suggests the limits of resistance, or agency on the part of migrants as highlighted by Ncube and Mkwananzi (2020). As Death (2016) argues counter-conducts are acts of resistance and not to be confused with political revolts.

While programs for integration are not strongly established in the global South, the South African government for example, in its White Paper on International migration suggests the need for the specific development of programmes that will facilitate the integration of migrants in the host society (Department of Home Affairs, 2017). The utility of the intersectional approach used in this study in identifying specific needs of migrants with respect to integration is important.

## Methodology

The research explores the labor market experiences of tied female migrants who emigrated to South Africa in the context of family migration. These spouses either accompanied their husbands or followed them for the purposes of family reunification. Semi-structured interviews were used, which are considered the most effective interview style to capture and document multiple experiences, explore contested issues, and capture multifaceted views of a phenomenon (Brinkman, 2014). Although 13 interviews were carried out in total, as part of a broader study, the narratives of six participants were included in this study, for the sake of focusing on labor market experiences, which was in conformity with the qualitative sample size recommendations by Braun and Clarke (2013). The inclusion criteria of the interviews used in the analysis of this study also considered the study by Guest et al. (2006), which found that 94% of most prominent or frequent codes emerge in the first six interviews and 97% by 12 interviews, thus implying data saturation. These six were chosen based on correct documentation required to work in South Africa (either work permit or permanent residence) and the relevance of their exposure in the workplace for the analysis at hand.

### Research Approach and Philosophy

A narrative, qualitative approach was used to garner the rich interpretations of reality and understanding of the world through the lens of the respondents. The emic approach used was regarded as suitable to understanding lived experiences from an intersectional perspective (Atewolougun, 2019). In addition, unlike the quantitative approach, the narrative approach is lauded for its ability to make the invisible visible (Meares, 2010).

Further, this study employed an interpretivist ontological research paradigm, which acknowledges that multiple realities or truths are the products of human subjectivity (Harrison, 2014). Through the voice of the participants, the study sought to understand or interpret certain phenomena.

From the interpretivist paradigm, reality is shaped by experiences and therefore becomes something to be interpreted. Through the narrative approach and interpretivist paradigm, the researchers scrutinized the lived experiences of others and interpreted them within a particular historical and social context.

### Data Collection

Purposive sampling was used to select respondents meeting the inclusion criteria to participate in the study. The advantage of purposive sampling is that it allows the researcher(s) to choose cases from which the most can be learned (Merriam, 2014). Thereafter, a snowball approach was utilised to complement the initial purposive sampling, in which participants were asked to refer other participants to the researchers, who met the inclusion criteria for the research.

Participants were invited to participate in one-on-one interviews *via* Zoom. The interviews began with an open-ended question designed to elicit rich narratives of their labor market experiences from pre to post migration. A semi-structured guide was subsequently used to interrogate any areas that were regarded as requiring further elaboration. Data collection took place over the period August 2020 to February 2021. Interviews were conducted over Zoom and recorded to an external device.

Upon referral to the researchers, with permission, the interviewees received a call explaining the nature of the research project and key ethical issues regarding consent and voluntary participation. After verbally consenting to be interviewed, each interviewee was furnished with a consent letter to sign which was to be returned prior to the date set for the interview. Owing to the predicted length of the interview, respondents were asked to choose a date and time which was most suitable and for which they would be available for the interviews. A review of the issues of consent also took place prior to the actual interview online.

### Data Analysis

This research employed thematic analysis to explore the narratives of accompanying spouses. The advantage of using thematic analysis is that it is not tied to any theoretical framework and therefore remains flexible mainly for use in various contexts (Braun and Clarke, 2006). As stated by Merriam (2014), in qualitative research the process of data analysis and data collection occur simultaneously. The six stages of thematic analysis by Braun and Clarke (2021) were employed to analyze the data, namely, getting familiar with the data through immersion in the data, transcribing the data, generating initial codes, review of themes, definition, and review of themes to ensure accuracy and alignment, and lastly, compiling the report using comparisons between the study’s results and extant literature.

Transcription of the audio-recorded interviews was done as soon as possible after each interview by playing back and listening to the recordings while typing the narratives word-for-word in a Microsoft Word document. This provided the researchers an opportunity to engage with the text and develop initial ideas about coding. An inductive approach to coding was used, allowing themes to emerge organically, within the broader context of the intersectionality theory. During the process of coding, notes were made in relation to key ideas for codes emerging from the text. Full transcriptions were subsequently loaded to ATLAS.ti for analysis. After a final decision on codes, themes were developed by grouping codes together in a way that answered the key questions arising from the study (Braun and Clarke, 2021). The verbatim quotes that were generated were used to substantiate the emergent themes in the results section.

### Research Ethics and Authorization

The study considered the main ethical considerations of informed consent, voluntary participation, academic integrity, and confidentiality. Owing to the risk of emotional distress arising due to the personal nature of some of the questions, access to psychological counseling was offered to all participants. Ethical clearance was sought from the General and Human Research Ethics Committee (GHREC) of the University of the Free State. Ethical Clearance Reference Number: UFS-HSD2020/0123/0506.

## Results

Utilizing the intersectional approach and the theory of governmentality, this study sought to understand the labor market experiences of tied migrants in South Africa. The study found that tied migrants were most likely to be subject to immobility, informality and precarity within the South African labor market and this were on account of their gender, race, ethnicity, migrant status but also on account of their locality and the various intersections thereof.

**Figure 1:**
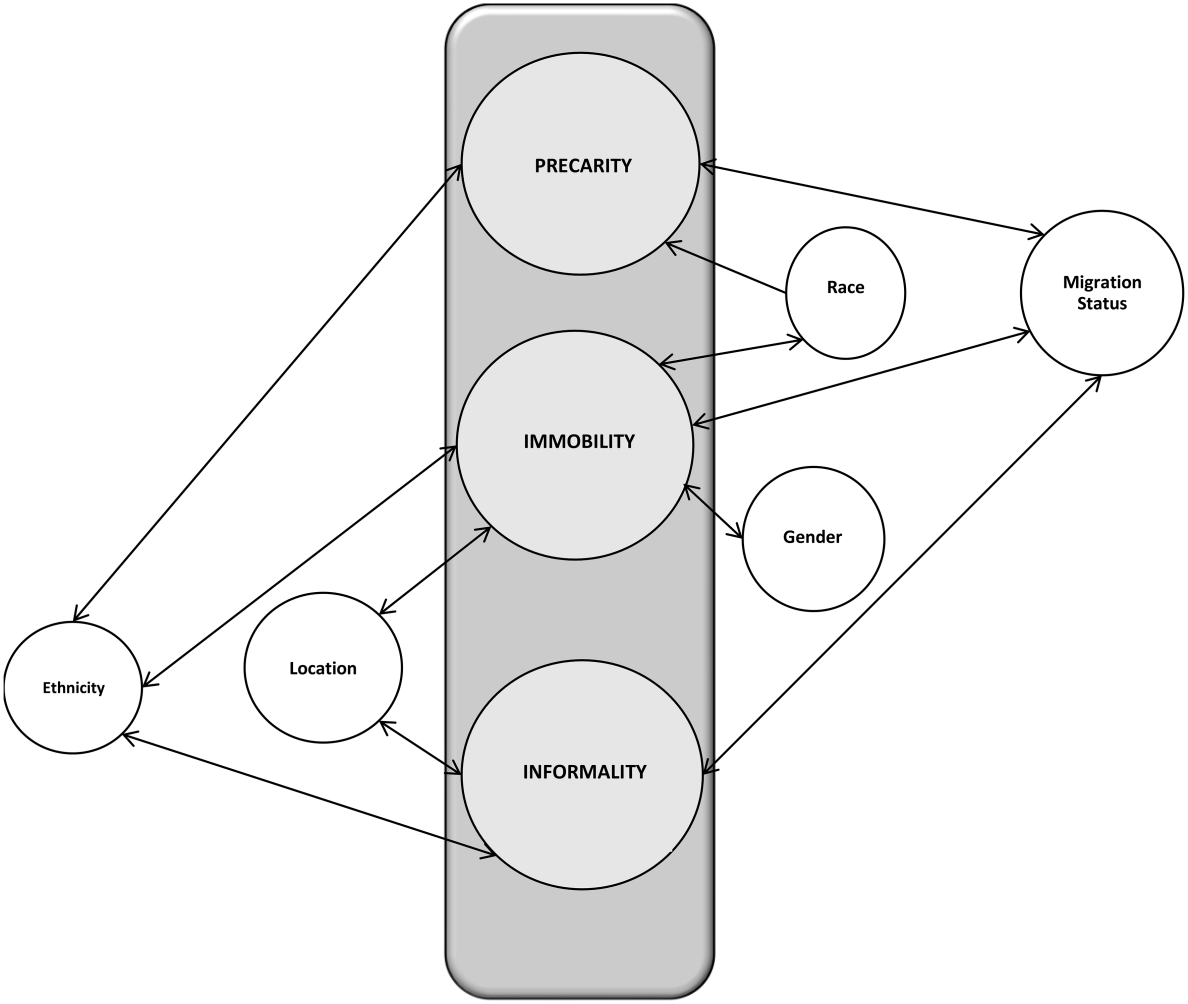
Interrelationship between subjectivities and intersections (author’s own illustration).

The term immobility does not refer only to physical immobility but is also concerned with subjective notions of progressing in one’s life. Spatial immobility represents being forced to remain in a particular position. Socioeconomic immobility refers to inability to earn a living because of one’s inability to get a job (O’Neil et al., 2016). Precarity refers to a state of vulnerability in which one’s agency can become limited (Hudson et al., 2019). Informality relates to the type of work which is not subject to national labor laws, tax, social protection nor entitlement to various employment benefits like sick leave or severance pay (Figure 1; OECD/ILO, 2019).

### Theme 1: Ethicized Ascriptions

Identifying as a non-South African was perceived to be one of the primary reasons for severely curtailed access to the South African labor market.

Strict employment legislation meant some opportunities for employment were deemed inaccessible to migrants because they were exclusively reserved for South African nationals. This was attributed to national policy. For instance, specific reference was made to the BBBEE policy, as supported by the following:

*Some of the jobs, they really state they want a South African citizen… You just have to accept they need someone with the credentials of being a citizen because the moment they say BBBEE, they want someone who is African and the African they want is a South African citizen* (Lucille, B (Honours) Accounting, lecturer, took 4 years to achieve LMI).

Ethicized ascriptions rooted in policies enacted at the institutional level, similarly, were also regarded as problematic. In institutions with such policies there appeared to be the wholesale discounting of qualifications and/or the working experience of foreigners. This made it particularly difficult for tied migrants to compete based on skills, qualification, or experience. This contributed to feelings of immobility, being overtaken, and left behind, as expressed by the following participant:

*After I had my permanent residence, I was employed as a primary school teacher but as an SGB* [School Governing Body] *teacher, I could not be employed in the Education Department because the policy is that they start by employing South Africans and there was nothing amazing for me in having a masters because they said the basic qualification for one to teach in their primary schools is a first degree* (Monica, PhD in Education Management, educationist, took 5 years to achieve LMI).

Ethnicity also intersected with race in giving rise to immobility. Notions of deservingness and acceptance in the labor market were linked to hierarchical, racialized, ethicized ascriptions. Being a black person from the African continent was regarded as a source for greater discrimination in comparison to black Europeans and black Americans. Being a black African migrant was therefore associated with increased propensity for broad exclusion from the South African labor market, as indicated by the following sentiment:

*Actually here, the culture is different* [regarding] *the acceptance rate of non-South Africans…It’s not super. They’d rather accept the Europeans, the Americans, no matter* [whether] *they are black skinned...* (Iris, PhD in Animal Science, lecturer, took 3 years to achieve LMI).

Tied migrants expressed difficulties with breaking into the professional labor market. This was attributed to beliefs about what kinds or types of jobs migrants should have access to. Access to professional white-collar jobs was viewed as limited owing to beliefs that foreigners were not suitable for such, as exemplified by the following:

[In Cape Town] *They believe foreigners should work in restaurants, or hotels as waiters and cleaners so, it’s hard to break into the* [professional] *work market* (Theresa-May, B Accounting, quality analyst, took 8 years to achieve LMI).

Despite being in possession of the correct documentation to work, being a foreigner was regarded as the basis for automatic disqualification by employers, regardless of one’s qualifications or experience. For the most part, tied migrants felt that there was no equality of opportunity to compete based on merit for work opportunities and were therefore, most likely to be discriminated and marginalized in this regard.

*And then you only realize if you know a few people that have applied for the same job that uh, but they took this person who is less qualified than me. Why did they do that? So somehow there’s this xenophobic thing as much as we run away from saying the word xenophobia, but they* [employers] *will be xenophobic* (Theresa-May, B Accounting, quality analyst, took 8 years to achieve LMI).

Overall deskilling of qualifications and experience was viewed as something that would happen time and time again, resulting in persistent occupational downward mobility, and poor upward mobility over the career trajectory even with change of employer. This was associated with feelings of being underemployed, underpaid, and feelings of immobility and lack of progress and general feelings of dissatisfaction, as shown in the following response:

*After 15 years of service [in education], I am employed as a new teacher. When I moved to the TVET college where I am now, I came in as an entry, despite 15 years of experience, plus five years acquired in South Africa. So, you start afresh. The salary is very low, lower than the least person. I am saying as long as I am employed, but am I getting what I’m supposed to be getting? No…. I feel I am very far from where I am supposed to be* (Monica, PhD in Education Management, educationist, took 5 years to achieve LMI).

And,

*I can work in any sector, that’s the good thing, but the job I do, it’s for a grade 10 or 12. It’s not something I would say I am passionate about. I’d be lying if I said I am passionate about it. It’s only that I can do it, there is no passion there* (Theresa-May, B Accounting, quality analyst, took 8 years to achieve LMI).

Precarity among tied migrants arose in the context of being unable to secure permanent work contracts. Instead, tied migrants found themselves confined to fixed term contracts wherein employers would not have to remunerate tied migrants at higher levels, as substantiated by the following:

*Of course, they are not spending much on me. So, I can remain there. They have refused to make me permanent. Yeah, I understand. I am not South African* (Iris, PhD in Animal Science, lecturer, took 3 years to achieve LMI).

As suggested in the quote above and supported by the quote below, tied migrants commonly experience poor remuneration levels and this is suggestive of the vulnerability to exploitation that tied migrants face in the labor market.

*I actually feel like I’m subsidizing my employer… The money that you earn cannot even take you through the whole month…Maybe a housemaid earns more than me* (Theresa-May, B Accounting, quality analyst, took 8 years to achieve LMI).

Persistent feelings of insecurity associated with one’s status as a foreigner, commonly experienced in South Africa in general were likely also experienced in the workplace. There was a strong sense among some that unfair dismissal on basis of ethnicity could arise at any given moment. Such views suggest a strong sense of precarity characterized by non-belonging, othering, and poor integration despite being employed in the labor market, as shown by the following:

*I am a foreigner in the company and it’s always like when they start speaking about unemployment, maybe one day, they’ll say, they’d rather employ a South African. You’re not fully secure cause you are a foreigner. It’s never like 100% sure* (Charlotte, Diploma in Journalism, administrator, took 7 years to achieve LMI).

And,

*We once had a cleaner at work who said [to me], “Oh, you are a kwerekwere* [derogatory term ascribed to black foreigners in SA]*.” It’s embedded in them this person is a foreigner, they do not belong* (Theresa-May, B Accounting, quality analyst, took 8 years to achieve LMI).

Derogatory comments were also associated with having a foreign accent and associated assumptions about one’s inability to communicate well, as supported by the following:

*But this older lady, I assume she had, or she has, you know, better experience but she actually came and said, but students may not understand us, you know these non-South Africans. We always have this kind of accent that students do not understand. Wow. It was so derogatory. And she was screaming, she wasn’t silent, people could hear.* [She said] *Students do not understand and that’s how students fail* (Iris, PhD in Animal Science, lecturer, took 3 years to achieve LMI).

### Theme 2: Migrant Status

Tied migrant status was the biggest impediment to entry into the labor market owing to the institutional rules making it illegal to work without the correct documentation. Tied migrant status critically, was also linked with career gaps which made it difficult to enter the formal labor market owing to limited work experience. Long gaps were also viewed as making tied migrants desperate for work and subsequently vulnerable to exploitation as evidenced by poor salaries, as substantiated by the following view:

*If people look at your CV and you have this gap. They do not even ask you whether you were studying. They’re looking for experience. Of which, where are you going to get experience if you are in university, that’s number one. And then when they see this employment gap, they actually think oh no this person is desperate. That way they capitalize. and to make it worse if you do not have correct papers, it’s rare to get paid well* (Theresa-May, B Accounting, quality analyst, took 8 years to achieve LMI).

Without permanent residence denoting legal authority to work, tied migrants resorted to informal, “piece” jobs. While permanent residence did not guarantee employment opportunities, it was regarded as instrumental to opening opportunities for formal, permanent, and professional jobs which was virtually impossible with tied migrant status. This is supported by the following:

*And then once I had the South African ID, it was easy to look for employment elsewhere, because now there was no restriction in terms of paperwork. As you know some companies, they know that with the accompanying Spouse Visa, you are not allowed to work. So, when you are trying to apply for new opportunities, they’ll just reject the application* (Andrea, Master’s in Development Studies, programme manager, took 5 years to achieve LMI).

And,

*You’re just accompanying your husband and that’s it. I did get some piece jobs, but you know how it is, it wasn’t really a formal thing. We actually have an ID now, a permanent residence* [permit]*. Then that’s when I got a formal job after that* (Charlotte, Diploma in Journalism, administrator, took 7 years to achieve LMI).

Interestingly, tied migrants with permanent residence rated their prospects of integration into the labor market to be below that of asylum seekers. Naturalization was viewed as opening a much broader range of opportunities which could not be accessed through permanent residence, as exemplified in the following:

*They* [the employers] *are not going to take you because they are forced to take South Africans or someone whose got citizenship, or even an asylum person I think they can take but not someone who has got a PR. No, they will not take someone with a PR. That’s SA for you* (Theresa-May, B Accounting, quality analyst, took 8 years to achieve LMI).

And,

*Remember, some of the jobs they really state that they want a South African citizen not a permanent resident. Some of the job adverts, you know for sure they’ll say, we want a citizen not a permanent resident* (Lucille, B (Honours) Accounting, lecturer, took 4 years to achieve LMI).

Having permanent residence was regarded as significantly useful in terms of facilitating access to formal employment and as instrumental in expanding employment options (job changes) however, it did not guarantee freedom from impacts of ethicized ascriptions, as expressed in the following:

*After I had my permanent residence, I was employed as a primary school teacher but as an SGB teacher. I could not be employed in the Education Department because the policy is they start by employing South Africans* (Monica, PhD in Education Management, educationist, took 5 years to achieve LMI).

### Theme 3: Gendered Ascriptions

Poor career progression in the workplace particularly with respect to promotion was strongly linked to gender. Greater challenges with advancing up the corporate ladder appeared more male dominated industries and in work environments with a strong patriarchal leaning.

*For me it’s two things happening, it’s being a foreigner and being a woman. It seems the South African education system is patriarchal in terms of leadership, in terms of promotion. For me it’s worse because I’m a foreigner* (Iris, PhD in Animal Science, lecturer, took 3 years to achieve LMI).

And,

*Being a woman in IT (Information Technology), the promotion just wasn’t coming* (Andrea, Master’s in Development Studies, programme manager, took 5 years to achieve LMI).

### Theme 4: Racialized Ascriptions

Identifying as Black was associated with poorer outcomes in comparison to other racial groups. For instance, being passed for promotion, getting a poorer salary or unfair treatment. Tensions arising from racialized ascriptions were apparent between various races including blacks, whites, colored and Indians, as exemplified in the following views:

*If they would hire a white person, we* [the blacks] *would train them, next thing the white person is promoted, and you are not* (Andrea, Master’s in Development Studies, programme manager, took 5 years to achieve LMI).

And,

*You’ve got so many battles to fight. It’s whites against blacks. There’s Indians against blacks. And I’m not saying this because it’s only the company, it’s just in general. I feel that there’s racism. In SA, you fight so many battles, you are black, you are a foreigner and you are a woman. Those are the three you are constantly fighting* (Charlotte, Diploma in Journalism, administrator, took 7 years to achieve LMI).

### Theme 5: Locality

One’s location in South Africa impacted significantly on what opportunities were available. Smaller towns were seen as having limited professional job opportunities and not having jobs in line with qualifications obtained in one’s home country. For most tied migrants, strong family ties made it difficult to move to other locations to take advantage of job opportunities there. Locality was therefore associated also with a lack of mobility, as supported by the following:

*For about three years we lived in Pretoria. I tell you, I did not realize I was a non-South African. Their criteria was just, can you do the job? Do you have this qualification, before we can take you, you must have your qualification in so and so sector. That was just only the criteria. Then you’ll be looking at the person interviewing are you not going to ask me if I’m a South African. Cannot you decipher from my tone? From my accent? But they do not care. Because they have mixed with a lot of people who are non-south Africans so me being a non-South African is not an issue so long as you have a permit book. It a totally different experience from what is obtainable in the Free State* (Iris, PhD in Animal Science, lecturer, took 3 years to achieve LMI).

And,

*I had got something at Rhodes, but family ties also came in, unfortunately… Something was coming up but eish, when we thought of it, the travelling and leaving the kids…It’s like, my husband feels we should always be together everywhere. I also feel the same, but I was tempted because I was looking at the salary and I was also looking at the upward mobility and the opportunity that had just been presented but he said money is not everything, we need family* (Iris, PhD in Animal Science, lecturer, took 3 years to achieve LMI).

Access to the labor market was predicated on language ability although this was more salient in certain localities compared to others. In this regard ethnicity connected with locality to produce relatively poor labor market outcomes. In these localities, lack of knowledge of Afrikaans, or any one of the indigenous languages was regarded as a factor diminishing the chances of gaining access into the labor market, as expressed in the following quotes:

*I have seen adverts where they want an Afrikaans speaking person to teach something in English, because they will have Afrikaans speaking people. The belief is that the students must be able to express themselves, so it’s a big limitation* (Lucille, B (Honours) Accounting, lecturer, took 4 years to achieve LMI).

The importance of this for LMI was reflected in that certain cities were regarded as more multi-cultural and accepting of foreigners, whereas some were regarded as less accepting of foreigners and with very few employment prospects. Other towns were considered more favorable in terms of offering job prospects for accompanying spouses. This was supported by the following quotes:

*… but here* [Cape Town] *because it’s racist, and they believe foreigners should work in restaurants, or hotels as waiters and cleaners so, it’s hard to break into the work market... I always still feel Pretoria was a better city than Cape Town personally. Because the people are better. They are not too racist. They are more accommodating* (Theresa-May, B Accounting, quality analyst, took 8 years to achieve LMI).

*It’s a small town where there’s no media anyway. I could not even apply for any job. And at that time, like journalism in that town, I could not find anything… And then we moved because he also wanted a promotion and we moved in a plantation deep in Mpumalanga... It was even worse for me at that time because I was like, I’m not working. It caused a strain actually, at that time in our marriage. I felt as if I’m the only one who is sacrificing. I cannot get a job and we are even moving deep, deep in plantations. You are happy in terms of your career, but I am not, I’m just here now* (Charlotte, Diploma in Journalism, administrator, took 7 years to achieve LMI).

*You know how it is in small towns. If you are a professional, what work will you do? There are limited options in terms of professional jobs. It’s difficult to find good paying jobs in smaller towns. That’s why we ended up moving to Johannesburg* (Andrea, Master’s in Development Studies, programme manager, took 5 years to achieve LMI).

Different locations were perceived very differently in the minds of interviewees which some places viewed as being very accepting of foreigners while others were not so accepting, as supported by the following:

*The only difficult place that I found it very difficult to break through it was here* [in] *Bloemfontein. It was a very difficult one because one, the language, they cannot employ you if you cannot say, what is it, morning in Afrikaans* (Unarine, Bachelor’s in HRM, real estate agent, took 4 years to achieve LMI).

*I think it was in the Northern Cape…., and the guy called me and spoke Afrikaans. I was like, oh, sorry, I do not understand Afrikaans. And he was like, are you for real? You do not understand Afrikaans? You want to work in this company? Oh, no, sorry. All their clients do not even speak English, so I should be able to speak Afrikaans. I just dropped the phone. For me that was like an unspoken code. You do not come here if you cannot* [speak Afrikaans]*. Then, there was a place in Bloemfontein, because you know Free State is just an Afrikaans zone no matter the Sothos and the Tswanas. You guys are just wasting their time if you do not know Afrikaans*... (Iris, PhD in Animal Science, lecturer, took 3 years to achieve LMI).

*No, I think Cape Town is naturally racist. So, the coloured people, they also have to look after their own which is very commendable because with us the black people we do not look after our own* (Theresa-May, B Accounting, quality analyst, took 8 years to achieve LMI).

## Discussion

Recent statistics show that South Africa is one of the top 20 nations globally among those hosting the largest populations of international migrants. The proportion of international migrants as a percentage of the population rose from 2.2% in 2000 to 7.1% in 2017 (United Nation, 2017). This data reflects South Africa’s prominence as a regional migration hub in sub-Saharan Africa (Mbiyozo, 2018) and its influential pull factors of a higher standard of living, relatively stable economy, and lower cost of living (Dinbabo and Nyasulu, 2015).

The labor market experiences of tied migrants in this study were distinctly shaped by governing technologies operating at the macro, meso and micro levels and mediated mainly through the gender, race, ethnicity migrant status and locality and the various intersections thereof. This study found that tied migrants were likely to be subject to immobility, precarity and informality in the South African labor market. Furthermore, this study affirmed the assertions that the exercise of power can result in differential treatment based on identity. Contrary to assumptions that high human capital fosters mobility and integration into the labor market (Khattab et al., 2020), this study confirms that tied migrants are in fact a unique group who despite their human capital, generally face unemployment, underemployment and generally, relatively poor integration into the labor market (Gerber and Wanner, 2019; Riaño, 2021).

Like findings by Riaño (2012), this study found that tied migrants face significantly diminished opportunities to integrate into the labor market. This, despite having secured the correct visas that allow them to work and having the appropriate qualifications and/or work experience. Where they may have integrated into the labor market, tied migrants in this study were likely however to be employed below their skill, underpaid, or kept on fixed term work contracts. In part, broad exclusion could be attributed to the very strict employment protection policies which pose significant barriers to LMI by migrants (Ballarino and Panichella, 2017; Ncube et al., 2020). For instance, the Broad Based Black Economic Empowerment [BBBEE] Policy (2013 as amended) represents one of the policies that perform a gatekeeping role in the labor market. The BBBEE sets out to redress the inequalities brought about by the Apartheid era by ensuring “equitable representation in all occupational categories and levels in the workplace,” (Government Gazette, 2013, p. 2). BBBEE appointments are solely reserved for Africans, Coloreds and Indians by birth or descent or those who became naturalized citizens prior to 1994, or for those who were eligible prior to 1994 but only became naturalized thereafter. Most of the migrants in this study arrived in South Africa post 2004 and therefore would not be eligible given the criteria set out in the BBBEE thus narrowing the opportunities available to them.

South Africa abounds with controversies about the purported impacts of migration on the labor market and the country’s scarce resources. Rising unemployment accompanied by the belief that migrants “steal jobs” and the alleged readiness of unskilled migrants to work for lower wages have all contributed to the rise of xenophobic attacks against immigrants (Landau, 2011; Chinyakata et al., 2019). These attacks are particularly severe against migrants of African and Asian origin (OECD/ILO, 2018). Anti-immigrant sentiments continue to be pervasive and have been expressed by prominent politicians including the former health minister Aaron Motsoaledi, who was quoted in the media as stating that foreign nationals are placing a burden on the South African health system (South African Broadcasting Corporation News, 2018). This study found that ethicized (us and them) ascriptions played the most significant role in impacting the labor market experiences of tied migrants in South Africa and through which tied migrants were subject to informality, immobility and precarity. This is not surprising however, given the lingering effects of South Africa’s apartheid history and the broad anti-immigrant sentiment that remains widely prevalent in the post-1994 era. As Souza and Flippen (2020) note, labor market experiences in South Africa are significantly shaped by socially constructed ethnic and racial boundaries.

This study suggests that ethicized ascriptions in addition, were likely not only to give rise to difficulties integrating into the labor market, but also dictating to some extent what types of jobs tied migrants might be able to access. Owing to these ascriptions tied migrants were likely to face lesser barriers to accessing work in the informal sector as opposed to the professional white collar labor market. This is consistent with the study by Souza and Flippen (2020), which suggests that male immigrants face formidable barriers in accessing the formal sector in South Africa. Consequently, it is not surprising that skilled female migrants would be subject to the same (Ncube et al., 2020; Vanyoro, 2021).

Most significantly, this study suggests the existence of wholesale deskilling, solely based on ethnicity. Ethicized downskilling was associated in non-equality of opportunity, significant down occupational mobility accompanied by poor prospects of upward mobility over time and across employers. For many tied migrants, career progress appeared non-existent and most appeared dissatisfied with their experiences in the labor market.

Many studies point to the difficulties of transferring institutional cultural capital from the country of origin as a significant barrier to labor market integration (Liversage, 2009; Ressia et al., 2017). In the South African context however, as suggested in this study, it appears it is difficult to transpose institutionalized cultural capital gained in the host country. This is contradictory to Bourdieu’s theory of capitals which presupposes that institutional cultural capital gained in the host country translates to greater advantage and ease of access into the labor market.

In this regard, institutional cultural capital gained in the host country does not appear to improve the chances of getting employed. It also does not provide a guarantee of full labor market integration nor prevent occupational downward mobility accompanied by underutilization of skills and poor pay owing to ethicized ascriptions. This is also suggestive of the prevalence of significant brain waste among skilled tied migrants. While this may be the case, it does not suggest that the acquisition of institutional cultural capital is not beneficial, however, it seems to be significantly more useful when it is acquired in areas of skills shortages which most of the tied migrants in this study did not have.

Precarity associated with ethicized ascriptions was also to be associated with vulnerability to exploitation, fears of being summarily dismissed, feeling of lack of security and non-belonging. Precarity was also associated with tied migrant status particularly in so far as it was associated with piece jobs that did not provide decent work protections for tied migrants. Critically, the career gaps (of up to several years) associated with tied migrant status was likely to result with consequences impacting on the entire career trajectory including vulnerability to exploitation evidenced by poor salaries. As previous studies have pointed out, it is difficult to recover loss resulting from downward occupational mobility over time.

As noted by Vanyoro (2021) identity markers can shift over time and space and this is true regarding tied migrants. As shown in this study, tied migrant status was strongly linked to what job one might be more eligible for. Acquiring legal permissions to work (for example *via* attainment of permanent residence or work visas) did not necessarily provide any form of advantage in terms of labor integration owing to racialized and ethicized ascriptions but it was viewed as important in allowing access to more formal work. In confirmation of the findings of Vermaak and Muller (2019), this study also confirms that barring the impacts of ethicized ascriptions, naturalized citizens may in fact have greater advantages in the labor market but perhaps only in so much as it opens a wider range of employment opportunities than other migrant statuses. Interestingly, this study also suggests that asylum seekers may fare better regarding integrating into the labor market as compared to tied migrants, i.e., (type of) documentation determines possibilities (Vanyoro, 2021).

The significance of the impact of gender on migration cannot be understated. Critically for example, female migrants almost inevitably are almost always relegated to the position of accompanying partner on account of their gender (Raghuram, 2004; Cooke, 2007; Fobker and Imani, 2017). Regarding labor market experiences, in this study surprisingly, gender appeared to only gain salience regarding male dominated, patriarchal work contexts and in the context of promotion opportunities. Prior research suggests that gender care responsibilities may be a significant barrier for female migrants in respect to LMI (Fobker and Imani, 2017; Ressia et al., 2017), however, this was not as distinct among tied migrants in this study. This may speak to the lesser import of traditional gender role beliefs among younger more educated couples who have divorced themselves from hegemonic norms. Alternatively, this may speak to the demands for migrant families to ensure socioeconomic wellbeing by having both partners well integrated into the labor market. In addition, migrant families in this context may have relatively fewer challenges in establishing a support network and accessing childcare for their younger children, which is deemed a significant barrier in other contexts (Cooke, 2007). Further, the relatively surprising low impact of gendered ascriptions in this study, however, may additionally point to the fact that many in this study did not have male-oriented critical skills which present opportunities to work in typically male dominated contexts, which also presents evidence of gendered inequalities.

In identifying locality as a mediating factor, this study similarly concurs with Vanyoro (2021) who suggests that identity axes are not the only mediating factors to consider regarding intersectionality. Locality became a strong mediator of labor market experiences owing to the structure of the labor market. As Statistics South Africa (2018) suggests, certain localities are more favorable for migrant labor market integration and mobility is favorable for LMI. As Föbker (2019) asserts regional labor markets and the availability of appropriate employment are critical to LMI. Language skills among tied migrants are key (Ballarino and Panichella, 2017), and in this study, language requirements were also strongly linked to locality.

Racialized ascriptions were regarded as bearing stronger influence in certain localities. Racialized ascriptions typically transcended the typical Black/White binary commonly associated with race and manifested in the differential treatment of blacks from coloreds and Indians as well. Racialized ascriptions were likely to be associated with career immobility wherein others of other races made faster progress up the corporate ladder.

Precarity in this regard, arose from the propensity for poorer salaries or unfair treatment. In addition, regardless of migrant status, in this study, family ties appeared to continue to play a significant role throughout the labor market trajectory of tied migrants (Ballarino and Panichella, 2017), contributing specifically to spatial immobility and therefore also significantly impacting on labor market experiences. Family ties made it difficult for tied migrants to access other employment opportunities in other regions. Limited mobility contributed significantly to the uptake of informal work particularly within smaller towns in South Africa.

Owing to ethicized ascriptions tied migrants faced non-equality of opportunity. This is in keeping with the notion that migrants generally face hierarchical ascriptions (Adjai and Lazaridis, 2013). The distinct presence of notions of othering or non-belonging in the workplace were linked to discriminatory and exclusionary practices (Riaño, 2021) and a significant level of poor integration into South Africa as a whole.

## Conclusion

Using the intersectional approach and the theory of governmentality this study found that tied migrants are likely to experience informality, immobility and precarity in the South African labor market. Various intersections of ethnicity, migrant status, race, gender, and locality were likely to be the greatest source of disadvantage leading to generally poor labor outcomes. Three key findings emerged from this study and that is, the presence of broad ranging ethicized deskilling is one of the main factors contributing to unemployment and underemployment, there are very limited returns to institutional cultural capital gained in South Africa, and that the attainment of the legal right to work, is not sufficient in terms of guaranteeing full labor migration. This suggests the need for targeted interventions if high human capital bearing female tied migrants are to become significant contributors to economic development in South Africa. Significantly, this study was able to contribute to the literature in terms of understanding how tied migrants fair in the South African labor context, which as Föbker (2019) asserts, is an important policy question.

## Data Availability Statement

The data that supports the findings of this study is available on request from the corresponding author. The data is not publicly available due to privacy or ethical restrictions.

## Ethics Statement

The studies involving human participants were reviewed and approved by the University of the Free State, General Higher Research Ethics Committee. The patients/participants provided their written informed consent to participate in this study.

## Author Contributions

This article was adapted from the doctoral thesis of FZ, who executed the research, while MS was the study leader and provided conceptualization guidelines and editorial inputs. All authors contributed to the article and approved the submitted version.

## Funding

This study was executed through personal funds.

## Conflict of Interest

The authors declare that the research was conducted in the absence of any commercial or financial relationships that could be construed as a potential conflict of interest.

## Publisher’s Note

All claims expressed in this article are solely those of the authors and do not necessarily represent those of their affiliated organizations, or those of the publisher, the editors and the reviewers. Any product that may be evaluated in this article, or claim that may be made by its manufacturer, is not guaranteed or endorsed by the publisher.
